# Pennsylvania Board of Examiners

**Published:** 1897-03

**Authors:** 


THE Pennsylvania State Board of Veterinary Medical Examiners held their midwinter examinations on December 15th and 16th, at Philadelphia and Pittsburg. Some four candidates presented themselves for examination, but one of this number had not been examined before; the others were of those who failed in April and June—the new applicant, Dr. R. M. Phelan, of Sharon, Pa., a graduate of Ontario Veterinary College, class of 1895. The diploma and Regents' certificate of Dr. Major Schofield were presented, but were not accepted by the Board, owing to the fact that his license of New York was granted under an exemption clause of the Act, and not by examination. He was directed to present himself for examination. But two examinations will be held hereafter, in June and December of each year. The Board awarded licenses to Drs. M. J. Chrisman and R. M. Phelan.

